# Efficient Delivery of Curcumin by Alginate Oligosaccharide Coated Aminated Mesoporous Silica Nanoparticles and In Vitro Anticancer Activity against Colon Cancer Cells

**DOI:** 10.3390/pharmaceutics14061166

**Published:** 2022-05-30

**Authors:** Chennan Liu, Fangyuan Jiang, Zifeng Xing, Lihong Fan, Yuan Li, Shaoning Wang, Junhong Ling, Xiao-Kun Ouyang

**Affiliations:** 1 School of Food and Pharmacy, Zhejiang Ocean University, Zhoushan 316022, China; cnl_082@163.com (C.L.); jfy0925@163.com (F.J.); xingzifeng@zjou.edu.cn (Z.X.); flihong2022@163.com (L.F.); liyuan632022@163.com (Y.L.); 2 School of Pharmaceutical Engineering, Shenyang Pharmaceutical University, Benxi 117004, China; wangsn_spu@163.com

**Keywords:** pH-sensitive drug release, silica, brown algae oligosaccharides, curcumin, colon cancer cells

## Abstract

We designed and synthesized aminated mesoporous silica (MSN-NH_2_), and functionally grafted alginate oligosaccharides (AOS) on its surface to get MSN-NH_2_-AOS nanoparticles as a delivery vehicle for the fat-soluble model drug curcumin (Cur). Dynamic light scattering, thermogravimetric analysis, and X-ray photoelectron spectroscopy were used to characterize the structure and performance of MSN-NH_2_-AOS. The nano-MSN-NH_2_-AOS preparation process was optimized, and the drug loading and encapsulation efficiencies of nano-MSN-NH_2_-AOS were investigated. The encapsulation efficiency of the MSN-NH_2_-Cur-AOS nanoparticles was up to 91.24 ± 1.23%. The pH-sensitive AOS coating made the total release rate of Cur only 28.9 ± 1.6% under neutral conditions and 67.5 ± 1% under acidic conditions. According to the results of in vitro anti-tumor studies conducted by MTT and cellular uptake assays, the MSN-NH_2_-Cur-AOS nanoparticles were more easily absorbed by colon cancer cells than free Cur, achieving a high tumor cell targeting efficiency. Moreover, when the concentration of Cur reached 50 μg/mL, MSN-NH_2_-Cur-AOS nanoparticles showed strong cytotoxicity against tumor cells, indicating that MSN-NH_2_-AOS might be a promising tool as a novel fat-soluble anticancer drug carrier.

## 1. Introduction

Colon cancer is a cancer type with worldwide incidence and high morbidity and mortality [1,2]. With the continuous deterioration in people’s quality of life, westernization of dietary habits, and the imbalance in population structure, the incidence of colon cancer has been slowly rising. According to the American Cancer Society, colon cancer has an incidence of 10.2% and a death rate of 9.2% [2,3]. Nano-drug delivery systems (NDDS) are an alternative therapeutic method to treat malignant tumors [4]. The issue of improving the bioavailability of drugs and reducing toxicity and side effects has always been a research hotspot in drug delivery. Nano drug delivery systems are represented by polymers, nano-capsules, polymer micelles [5], liposomes [6], inorganic nanoparticles such as gold [7], graphene [8], mesoporous silicon [9], and polydopamine [10], have been widely used in tumor therapy. By endowing the common anticancer drugs with the specificity of targeted delivery, anti-tumor activity can be improved [11]. NDDS mainly rely on the forces between surface groups and molecules, including van der Waals forces and hydrogen bonds, so that drug molecules can be dissolved, adsorbed, or covalently bound to the surface of particles. Drugs can be wrapped or embedded in these systems to form a stable drug carrier [12,13]. The rapid uptake of nanoparticles by the reticuloendothelial system causes the carrier to circulate in the blood for a short amount of time to reach the minimum therapeutic concentration. NDDS with particle sizes ranging from 1 to 1000 nm systematically reach the tumor site and release their active drugs [14,15]. Stimulation-responsive functional NDDS target the release of drugs upon specific endogenous (pH, redox, ROS) and exogenous (light, heat, magnetic) stimuli that can be constructed, considering the acidic and reductive characteristics of the tumor cell microenvironment [16].

The pH of normal biological tissues is neutral, while the pH of the tumor microenvironment is slightly acidic: the pH value of tumor cells is 5.7–7.8 and that of tumor cell stroma can reach 4.5–5.5 [17]. Thus, the acidic environment around and inside tumor cells provides a triggering condition for pH-sensitive nano-carriers. Stimulation-responsive functional NDDS can achieve “zero release” of drugs under pH 7.4 conditions, which prevents the release of drugs before reaching the lesion site, while effective targeted release can be enabled under the weak acidic conditions of the tumor microenvironment [18,19]. Mesoporous silica nanoparticles (MSNs) are a type of porous non-metallic material carrier. Due to their high surface area, stability, targeting ability, biocompatibility, and many other excellent characteristics, MSNs have shown great application advantages in tumor-targeted drug delivery [20,21,22]. Researchers have modified the surface of MSNs to enable the loading of drugs into their pores [23,24]. Moreover, MSNs were encapsulated with appropriate “gating” molecules, producing a drug delivery system that can respond to various environmental changes such as pH, redox, enzymes, and temperature [25]. Zhang et al. constructed pH-sensitive silica NDDS using human serum albumin, an endogenous protein, as a gating molecule [26]. Functional groups on the modified MSN surface can enhance targeted delivery through nanoparticle-environment interaction [27,28]. Different organic functional groups have been covalently or electrostatically attached to the MSN surface to achieve the ideal characteristics of MSNs [29]. Curcumin (Cur) is a natural polyphenolic compound extracted from turmeric and has antioxidant and anti-inflammatory effects [30,31,32]. Studies have shown that Cur can also exert anti-tumor effects by interacting with the transcription factor nuclear factor-kappa ß, and protein kinase C, eventually leading to tumor cell apoptosis [33,34,35]. However, the bioavailability and therapeutic index of Cur are low due to its poor solubility in aqueous solvents, which hinders its clinical applications [36,37,38]. Alginate oligosaccharide (AOS) is an acidic, low-molecular-weight polymer derived from alginates [39]. It is an oligosaccharide with weak toxicity, high biodegradability, and high solubility. At the same time, it has various biological activities, such as anti-inflammatory, antibacterial, immune-regulatory, and anti-tumor effects [40,41]. Some papers [42] have discussed drug delivery systems based on MSN encapsulation using functional polysaccharides, including chitosan, hyaluronic acid, sodium alginate, and dextran, to achieve drug release control. However, to the best of our knowledge, this is the first report on the use of AOS to coat MSNs. Since MSN-NH_2_ is positively charged, it can absorb the negatively charged Cur through electrostatic adsorption and can be linked with AOS through an amide bond. Therefore, we prepared a functional nano-drug carrier particle modified with AOS to achieve pH-responsive release of Cur, improving its stability and bioavailability, and accomplishing targeted Cur release in tumor sites. In addition, we studied the in vitro release characteristics, cellular uptake, and safety of this nano-drug delivery system.

## 2. Materials and Methods

### 2.1. Materials

Curcumin (Cur, >99%), alginate oligosaccharides (AOS, >99%) ethyl orthosilicate (TEOS, AR), potassium bromide (KBr, SP), coumarin–6 (>99%), cetyltrimethylammonium bromide (CTAB, >99%), cetyltrimethylammonium chloride (CTAC, >99%), 3-amino-propyl triethoxysilane (APTES, >99%), N-hydroxysuccinimide (NHS, AR), and 1-(3-Dimethylaminopropyl)-3-ethylcarbodiimide hydro (EDC, AR) were purchased from Shanghai Aladdin Company. Monosodium phosphate (NaH_2_PO_4_), disodium phosphate (Na_2_HPO_4_), dipotassium phosphate (K_2_HPO_4_), triethanolamine (TEA), sodium hydroxide (NaOH), hydrogen chloride (HCl), and ethylene glycol are all analytical pure chemicals acquired from Sinopharm Chemical Reagent limited corporation.

### 2.2. Preparation of MSNs

MSNs were prepared according to the methods in the literature, with several adjustments [43,44]. CTAB (0.2 g) was dissolved in 96 mL of water, and the mixture was heated and stirred. When the temperature reached 80 °C, 0.7 mL of 2 M NaOH solution was added to the CTAB solution and continued to be stirred for 30 min. Then, 1.4 mL of TEOS was slowly added to the solution, and the mixture was vigorously stirred at 80 °C for 2 h. After cooling, a white precipitate was obtained by centrifugation. Then, 10% (*v*/*v*) hydrochloric acid-ethanol solution was used to remove the template and 6 h after reflux to obtain the product, MSN-1.

CTAB (500 mg) was dissolved in 200 mL of deionized water, ethylene glycol (40 mL), and 1 M NaOH (3.5 mL). The mixture was heated to 80 °C and stirred vigorously for 1 h. Then, ethyl orthosilicate (2.5 mL) was rapidly added to the solution, and the mixture was kept at 80 °C for 2 h until a white precipitate was formed. The precipitate was washed with water and ethanol at 10,000 rpm (20 min). After vacuum drying, the white precipitate was calcined at 550 °C for 6 h to obtain the product, MSN-2.

Next, 2 g of CTAC was dissolved in 20 mL of deionized water, and 0.32 mL of TEA was dropped into the solution, followed by stirring at 95 °C for 1 h. TEOS (1.5 mL) was then added slowly into the solution. The reaction was completed after 1 h. The products were washed with deionized water and ethanol three times, vacuum-dried at −60 °C, and calcined in a Muffle furnace at 550 °C for 6 h to remove the template and obtain MSN-3 [45].

### 2.3. Preparation of Amino MSNs

The amino modification method was performed as previously described [46]. Briefly, 1 g of MSN was reacted with 20 mL of APTES in 100 mL of toluene (24 h, 60 °C). At the end of the reaction, the materials were washed alternately with ethanol and water to obtain amino MSNs (MSN-NH_2_).

### 2.4. Preparation of MSN-NH_2_ Coated with AOS

**Figure 1:**
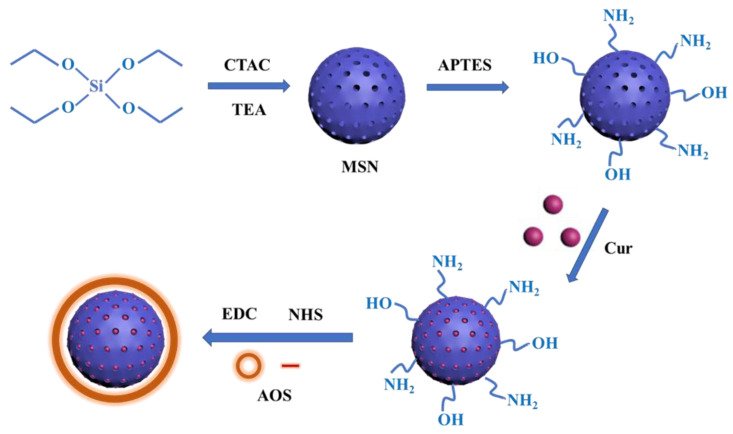
Preparation of MSN-NH_2_-Cur-AOS nanoparticles.

To prepare a 1 mg/mL Cur solution, 20 mg of Cur was accurately weighed and mixed with 20 mL of anhydrous ethanol. The Cur solution (1 mL) was mixed with PBS (10 mL) and 20 mg of MSN-NH_2_ and stirred at 25°C protected from light for 12 h. Subsequently, 50 mL of 1% (*w*/*v*) AOS solution was prepared, and a certain volume of AOS solution (20 mL) was mixed with 50 mg of NHS and 0.1 g of EDC, and the carboxyl group was activated by stirring in the dark for 4 h. Drug-loaded MSN-NH_2_ solution was added to the activated AOS solution, stirred at 25 °C without light for 12 h, and centrifuged (8000 r/min, 10 min) to obtain MSN-NH_2_ nanoparticles coated with AOS (MSN-NH_2_-Cur-AOS). Nanoparticles without Cur (MSN-NH_2_-AOS) were prepared using the same method. Figure 1 shows the schematic diagram of MSN-NH_2_-Cur-AOS preparation. The absorbance at 426 nm was detected using an ultraviolet-visible spectrophotometer (UV-Vis, U-2910, Thermo, Waltham, MA, USA) to calculate the loading concentration of Cur and the loading efficiency of MSN.

### 2.5. Characterization

#### 2.5.1. SEM and TEM

A scanning electron microscope (SEM, Hitachi S-4800, Tokyo, Japan) was used to evaluate the surface morphology of the nanoparticles. Under the acceleration voltage of 20 kV, the samples were properly magnified and observed, and clear and regular particle images were obtained. Transmission electron microscope (TEM, JEM-2100, Tokyo, Japan) images were obtained by Lorentz transmission electron microscopy. A proper amount of nanoparticle powder samples was dispersed on the conductive copper mesh for TEM scanning.

#### 2.5.2. FTIR

The composition of the functional group was analyzed by Fourier transform infrared spectrometry (FTIR, Bruker Tensor II, Bremen, Germany). The samples were fully mixed with KBr. The infrared spectrum scanning range was between 4000 and 400 cm^−1^, and the instrument resolution was set as 4 cm^−1^. Carbon dioxide (CO_2_) and water (H_2_O) peaks were subtracted from the original spectrum to obtain the final FTIR spectrum.

#### 2.5.3. TGA

Thermogravimetric analysis (TGA) was performed using the TAQ50 thermal analyzer (Shimadzu, Kyoto, Japan) at a heating rate of 10 °C/min under an N_2_ atmosphere, and the test interval was set to 37–800 °C.

#### 2.5.4. Zeta Potential and PDI

The zeta potential and Polymer dispersity index (PDI) were measured using a Malvern laser granulometer (ZS90, Malvern, UK): a few samples were prepared and dispersed with deionized water. Ultrasonication was performed for 15 min before measurements, and an appropriate amount of suspension was removed into a special sample pool.

#### 2.5.5. Size

The average particle size of the composite nanoparticles was determined using the dynamic light scattering (DLS) technology (ZS90, Malvern, UK). Before each measurement, the samples were diluted with water at the same pH to avoid multiple scattering. The samples were placed in a plastic cuvette for testing and the test results were recorded.

#### 2.5.6. BET

Brunauer–Emmett–Teller (BET, Tri Star II 3020, Micromeritics, Norcross, GA, USA) method is used to characterize parameters such as specific surface area, pore volume, and pore size of mesoporous materials. An appropriate number of samples were prepared for degassing and transferred to liquid nitrogen for adsorption-desorption analysis. The BET analysis was performed in an N_2_ atmosphere for 6 h, and the desorption temperature was set at 120 °C.

#### 2.5.7. XPS

The surface chemical composition and the chemical state of each component of the nanoparticles were analyzed using an X-ray photoelectron spectrometer (XPS, Kratos, Manchester, UK).

### 2.6. Encapsulation Efficiency (EE) and Loading Efficiency (LE)

**Figure 2:**
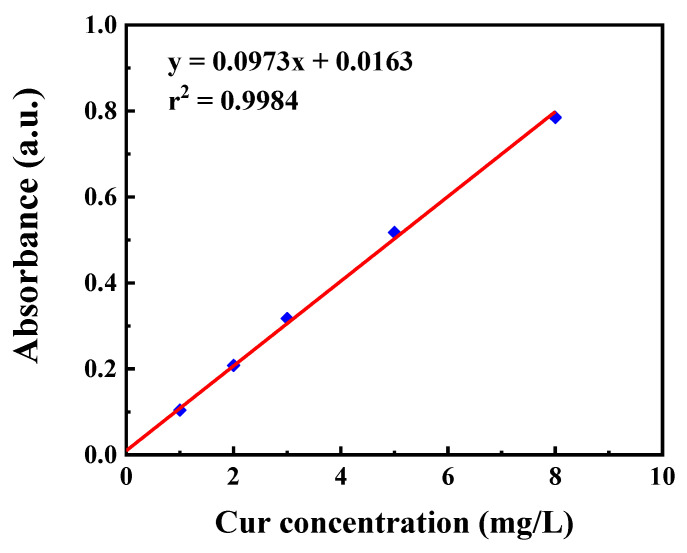
Standard curve of curcumin.

Encapsulation efficiency (EE) and loading efficiency (LE) are important indicators of the quality of nanomedicines, which respectively represent the efficacy of encapsulation and loading. Following the preparation of MSN-NH_2_-Cur-AOS, the supernatant was collected by centrifuging the nanoparticle samples (8000 r/min, 15 min). The mass of Cur initially added was denoted by *W_t_*. *W_d_* denoted the free Cur content in the supernatant, and *W_l_* denoted the dry mass of precipitate. The supernatant of MSN-NH_2_-AOS obtained by the same method was used as a reference. Stock solutions of Cur (200 mg/L) were diluted to obtain serial standard solutions to fit the standard curve (shown in Figure 2), and the content of Cur was determined using the standard curve equation and UV measurements at 426 nm. EE and LE of the drug were calculated according to the following formulas:

(1) $$EE=\frac{W_{t}-W_{d}}{W_{t}}\times 100\%\;$$

(2) $$LE=\frac{W_{t}-W_{d}}{W_{l}}\times 100\%\;$$

### 2.7. Release Kinetics

Five milligrams of MSN-NH_2_-Cur-AOS and MSN-NH_2_-Cur were added into a centrifugation tube containing 20 mL of PBS (pH 7.4 and pH 5.0) and placed in a 37 °C constant-temperature water bath oscillator (150 r/min) to investigate the in vitro release kinetics of the nanoparticles. At the time points of 1, 2, 4, 6, 8, 12, and 24 h, the sample was centrifuged, and 1 mL of the supernatant removed (and replaced with 1 mL of PBS solution at the original pH value), and then the samples were placed back into the oscillator to continue oscillating. At the same time, the release of Cur within 2 h was studied. The absorbance at 426 nm was measured using a UV spectrophotometer and the concentration of Cur in the solution was calculated.

### 2.8. Cytotoxicity

The cytotoxicity of drug-loaded nanoparticles in HCT-116 colon cancer cells (Provided by Zhejiang University) was evaluated using the MTT assay [44]. Briefly, HCT-116 cells were seeded on 96-well plates at a density of 5 × 10^4^ cells/well. After 24 h of culture, the cells were subjected to a DMSO water diluent (control) medium containing 10, 20, 30, 40, or 50 µg/mL free Cur (MSN-NH_2_-Cur-AOS), or medium containing the nanoparticles accounting for an equal amount of Cur (MSN-NH_2_-AOS). The cytotoxicity of MSN-NH_2_-AOS and MSN-NH_2_-Cur-AOS on L929 fibroblast cells (provided by the Chinese Academy of Sciences) was determined using the same method. L929 cells were seeded on 96-well plates at a density of 2 × 10^4^ cells/well, and after 24 h of culture, different concentrations of MSN-NH_2_-AOS and MSN-NH_2_-Cur-AOS solutions were added. Simultaneously, an equal volume of fresh culture medium was supplied to the blank control cells. After culturing the cells for 24 h, each well was supplied with 20 μL of MTT reagent solution. After culturing the cells in a CO_2_ incubator for 4 h, the supernatant was removed and 150 μL of DMSO was added. The samples were vortexed for 8 min before the test and OD values at 490 nm were measured using a microplate reader (Infinite 200 PRO, Tecan, Zurich, Switzerland). The relative cell viability (%) was calculated according to the following formula:

(3) $$\mathrm{Viability}\;\left(\%\right)=OD_{1}/OD_{2}\times 100\%$$

where *OD*_1_ denotes the absorbance value of the cells treated with the drug, and *OD*_2_ denotes the absorbance value of the blank control group.

### 2.9. Cellular Uptake

For cellular uptake experiments, we loaded MSN-NH_2_-AOS nanocarriers with coumarin-6 since Cur is prone to light pollution and coumarin-6 is similar to curcumin in structure. The preparation method was the same as that of MSN-NH_2_-Cur-AOS [47,48]. The uptake of MSN-NH_2_-Coumarin-6-AOS nanoparticles by colon cancer cells was investigated via fluorescence staining images. HCT-116 cells were seeded into a 6-well culture plate with an initial density of 1 × 10^6^ cells/well and cultured for 24 h. The cells were then treated as follows: First, two wells were supplied with MSN-NH_2_-AOS or MSN-NH_2_-Coumarin-6-AOS nanoparticles at an equal concentration and cultured at 37 °C for 30 min, while two wells were supplied with PBS as control; at the end of 30 min, an equal amount of free MSN-NH_2_-AOS or MSN-NH_2_-Coumarin-6-AOS nanoparticles were added into the remaining two wells and the plate was cultured for an additional 30 min under the same conditions. Next, the supernatant was carefully discarded, and the cells were rinsed 3 times with PBS, shaking slowly for 2 min each time. Finally, all cells were collected and analyzed for fluorescence intensity using an inverted fluorescence microscope (Leisi, Nikon, Tokyo, Japan).

### 2.10. Statistical Analysis

All quantitative data were obtained from at least three independent experiments and expressed as mean ± standard deviation (SD). Statistical comparisons were performed using a one-way analysis of variance (ANOVA). *p* values < 0.05 (*) and < 0.01 (**) were considered statistically significant.

## 3. Results and Discussion

### 3.1. Characterization of MSNs

**Figure 3:**
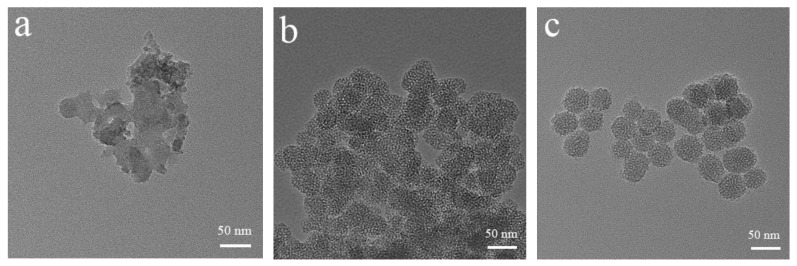
TEM images of MSN-1 (**a**), MSN-2 (**b**), and MSN-3 (**c**).

Three different methods were used to prepare MSNs and the structures of the products were compared. No porous structure was observed in MSN-1, but a rough surface, different particle sizes, serious agglomeration, and poor dispersion (Figure 3a). Compared with that of MSN-1, the particle size of MSN-2, which was prepared by removing the template by pickling, was slightly increased (Figure 3b). As shown in Figure 3c, MSN-3 nanoparticles had a regular spherical shape, a porous surface, and uniform sizes, and the surfaces were smooth. They had better dispersion than MSN-2 particles. The changes in particle size, potential, and PDI during the modification were further tested using a zeta-potentiometer. Table 1 shows significant differences in the particle sizes of MSN-1, MSN-2, and MSN-3, among which MSN-3 had the smallest particle size, and the surface potentials were measured as −28.3, −29.6, and −32.2 mV, respectively. PDI also confirmed that MSN-3 particles had better redispersal than the other two particles. Therefore, MSN-3 was selected for subsequent experiments.

### 3.2. Preparation of MSN-NH_2_-Cur-AOS Nanoparticles

**Figure 4:**
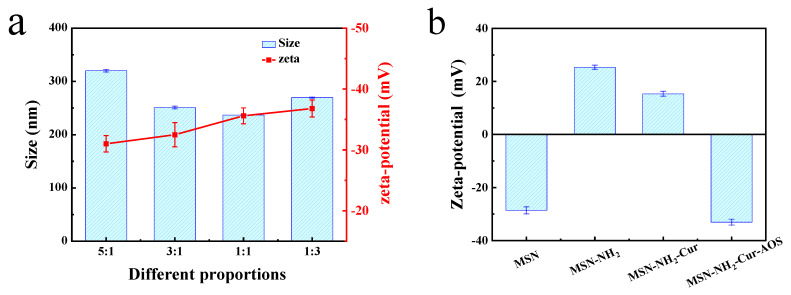

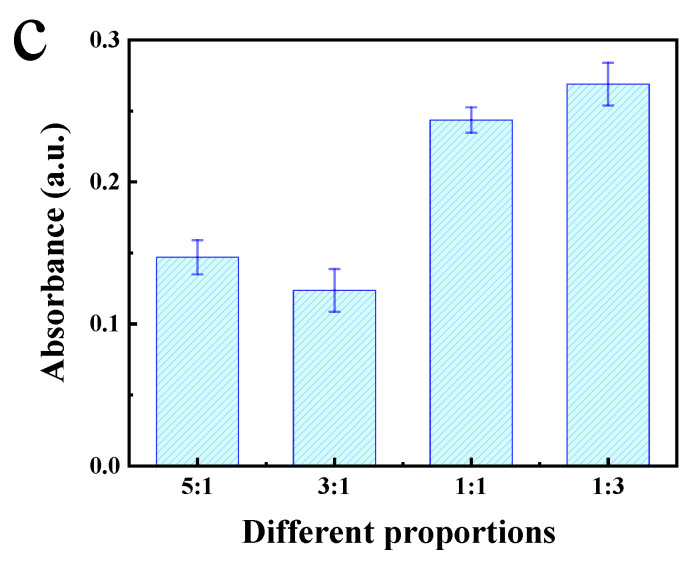
The particle sizes and zeta potentials of MSN-NH_2_-AOS prepared with different mass ratios of MSN-NH_2_ and AOS (**a**). Zeta potentials of MSN, MSN-NH_2_, MSN-NH_2_-Cur, and MSN-NH_2_-Cur-AOS (**b**). Absorbance of Cur released from MSN-NH_2_-Cur-AOS nanoparticles with different mass ratios after loading the same content of Cur (**c**).

MSN-NH_2_ exists as a cation in an aqueous solution, but AOS is in the form of an anion in the aqueous solution. Therefore, when MSN-NH_2_ and AOS are mixed evenly by mechanical stirring, they self-organize into nanoparticles through electrostatic interaction [49]. The solution of MSN-NH_2_ at a low concentration is transparent, with almost no flocculation. However, the solution containing a high concentration of MSN-NH_2_ results in aggregation and an enormous amount of precipitation. Therefore, the effect of the MSN-NH_2_ to AOS ratio on the morphology of nanoparticles needs to be investigated. As seen in Figure 4a and Table 2, when the ratio of MSN-NH_2_ to AOS in the solution was 5:1, because of the relatively high MSN-NH_2_ ratio, the amount of free MSN-NH_2_ remaining in the solution was high, and the nanoparticles obtained had a large particle size and poor dispersion. This may be due to the aggregation of excess MSN-NH_2_ cations, which destroy the particle dispersion steady-state and result in particle aggregation and particle size increase [50]. When the MSN-NH_2_ content was decreased (5:1 to 1:1), the size of MSN-NH_2_ nanoparticles decreased significantly (*p* < 0.05), and MSN-NH_2_ particles combined with AOS reached the dispersion state at the same time due to the positive and negative charge attraction. However, when the ratio of the two compounds was adjusted from 1:1 to 1:3, the particle increased in size from 236.80 ± 0.54 nm to 269.50 ± 1.03 nm, indicating that more AOS remained in the nano-dispersion solution. Meanwhile, the surface potentials of nanoparticles, which were all negative, continuously decreased with the decrease of MSN-NH_2_ content, indicating that AOS had a dominant influence on the potential of nanoparticles [51]. In addition, the zeta potential changed during the preparation of MSN-NH_2_-Cur-AOS. As shown in Figure 4b, the zeta potential of MSN increased from −28.6 ± 1.3 mV to 25.3 ± 0.8 mV after the preparation of MSN-NH_2_ by amine functionalization. After loading Cur and coating with AOS, the zeta potential of MSN-NH_2_ decreased from 25.3 ± 0.8 mV (MSN-NH_2_) to 15.3 ± 0.9 mV (MSN-NH_2_-Cur) and −33.1 ± 1.1 mV (MSN-MH_2_-Cur-AOS). The variation of zeta potential indicated that the modification of MSN was successful. Figure 4c shows the absorbance of curcumin released by the nanocarrier after loading with 0.5 mg of Cur. The absorbance of Cur increased with the increasing AOS content. While the AOS ratio increased from 1:1 to 1:3, the rising trend of the absorbance of Cur was slow. On this basis of these data, we selected the 1:1 ratio for subsequent experiments.

### 3.3. Characterization of MSN-NH_2_-Cur-AOS Nanoparticles

**Figure 5:**
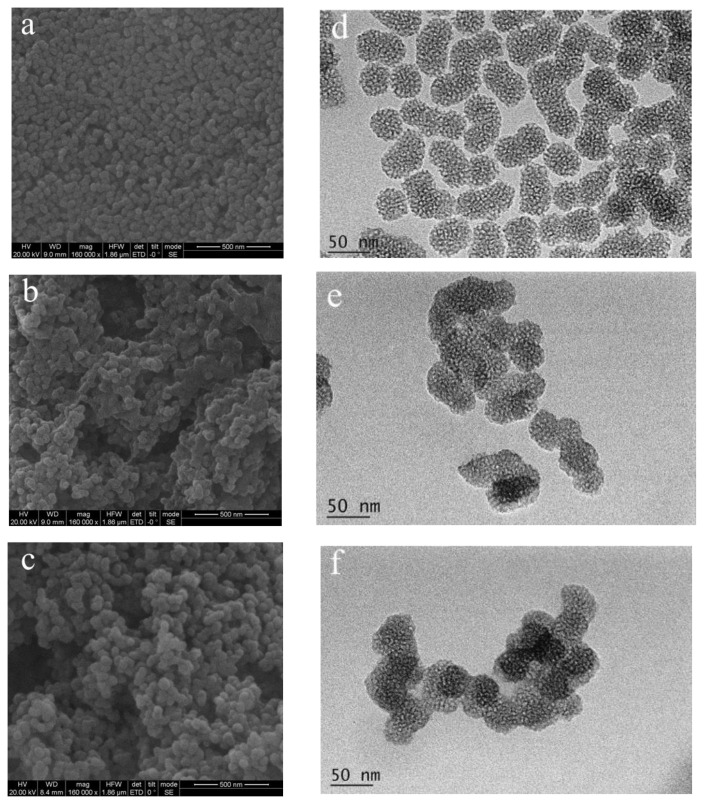
SEM images of MSN-NH_2_ (**a**), MSN-NH_2_-AOS (**b**), and MSN-NH_2_-Cur-AOS (**c**). TEM images of MSN-NH_2_ (**d**), MSN-NH_2_-AOS (**e**), and MSN-NH_2_-Cur-AOS (**f**).

As shown in the SEM image in Figure 5a, blank MSN-NH_2_ typically had a spherical structure with circular mesoporous pores [6]. Meanwhile, the particles clustered after the addition of AOS, as shown in Figure 5b, mainly due to the adhesion effect of the linear polysaccharide. Following the addition of Cur, the particle size of MSN-NH_2_-Cur-AOS particles increased, as shown in Figure 5c. After MSN surface functionalization with APTES, loading with Cur, and gradual conjugation with AOS, TEM images showed that amino-functionalized MSN nanoparticles had a porous mesopore structure. AOS can be observed around the nanoparticles, as shown in Figure 5d–f. Furthermore, the nanoparticle size increased significantly, the porosity decreased gradually, and the honeycomb arrangement could not be clearly observed. These morphological changes present additional evidence for the existence of AOS coating on the MSN surface. This aspect proves the successful construction of MSN-NH_2_-Cur-AOS [52].

**Figure 6:**
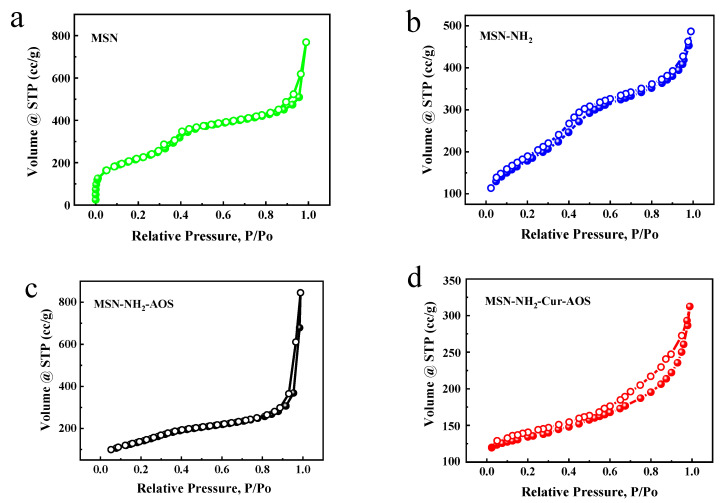
N_2_-adsorption and desorption curves for MSN (**a**), MSN-NH_2_ (**b**), MSN-NH_2_-AOS (**c**), and MSN-NH_2_-Cur-AOS (**d**).

N_2_ adsorption and desorption curves and parameters are shown in Figure 6 and Table 3. The specific surface areas of MSN, MSN-NH_2_, and MSN-NH_2_-Cur-AOS were 783.9, 648.1, and 286.3 m^2^/g, respectively, as measured using the BET method. The decrease in specific surface areas indicated that the surface of MSN nanoparticles had been successfully coated with AOS. To a certain extent, the specific surface area can reflect the smoothness of the surface of nanoparticles. In terms of specific surface area, the surface of MSN-NH_2_-Cur-AOS was the roughest, which was consistent with the results of SEM characterization. The pore size and pore volume of nanoparticles were analyzed by the Barrett-Joyner-Halenda method. After ATPES modification, the pore size was slightly reduced from 6.165 to 4.646 nm, indicating that a small part entered the MSN interior. Following AOS coating, the pore size of the sample decreased significantly (1.126 nm), which proved that AOS successfully encapsulated MSN. The decreases in specific surface area and pore size indicated that AOS could effectively block the mesoporous channels of MSN, which could help effectively encapsulate and store drugs and avoid drug leakage and premature release.

**Figure 7:**
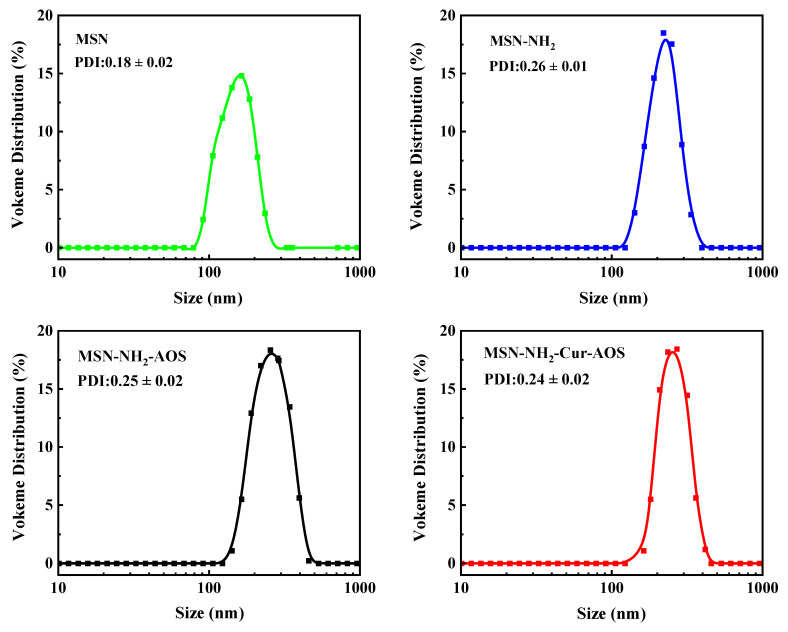
DLS graphs of MSN, MSN-NH_2_, MSN-NH_2_-AOS, and MSN-NH_2_-Cur-AOS.

The dimensions of MSN, MSN-NH_2_, MSN-NH_2_-AOS, and MSN-NH_2_-Cur-AOS nanoparticles were analyzed and measured using DLS [53,54]. Figure 7 shows the average particle size of MSN and MSN-NH_2_ was about 158 nm (PDI = 0.18 ± 0.02) and 227 nm (PDI = 0.26 ± 0.01), respectively, indicating the successful introduction of -NH_2_. The average particle size of MSN-NH_2_-AOS increased to 310 nm (PDI = 0.25 ± 0.02) when coated with AOS. It can be observed that Cur entering MSN pores had little effect on the particle size of the nanocarriers. Compared with MSN-NH_2_ nanoparticles, MSN-NH_2_-AOS and MSN-NH_2_-Cur-AOS nanoparticles had larger average sizes and wider size distributions, and there was little difference in size between the two nanoparticles. The above results demonstrated the successful preparation of MSN-NH_2_-Cur-AOS, the successful entry of Cur into MSN-NH_2_ mesopore, and the encapsulation by AOS mainly in MSN-NH_2_.

**Figure 8:**
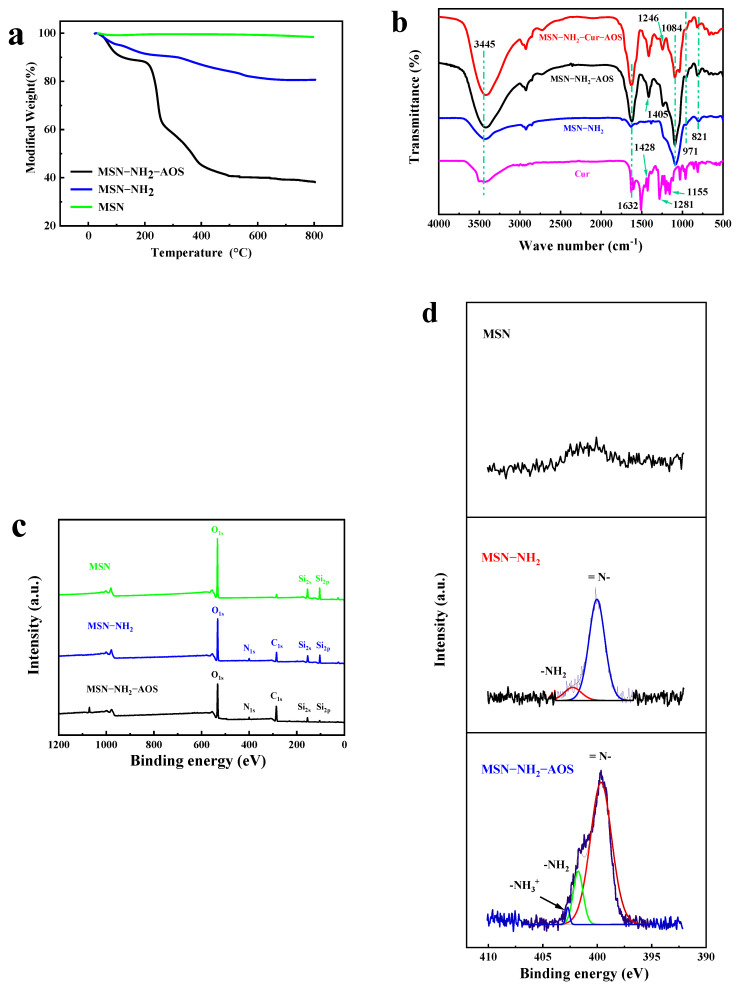
TGA of the nanoparticles (**a**). FTIR of the nanoparticles (**b**). XPS full spectrum of MSN, MSN-NH_2_, and MSN-NH_2_-AOS (**c**). N_1__s_ spectrum of MSN, MSN-NH_2_, and MSN-NH_2_-AOS (**d**).

Figure 8a shows the TGA curves of MSN, MSN-NH_2_, and MSN-NH_2_-AOS nanoparticles. The weights of the three kinds of nanoparticles were similar at more than 80% of the temperature range of 30–200 °C. In the test range from 30 to 800 °C, the weight loss of MSN alone was only 1.5%. Before 100 °C, the main loss was the binding water on the surface of MSN nanoparticles, which may be due to the dense mesopore structure of MSN. MSN-NH_2_ had a mass loss in the temperature range of about 20 to 100 °C and 400 to 630 °C, which was caused by the thermal decomposition of the grafted organic matter APTES and a large amount of combined hydrothermal volatilization, proving the success of grafting. In addition, the weight loss of MSN-NH_2_-AOS between 20–100 °C was caused by surface water separation, and the weight decreased significantly between 200–400 °C, which was related to the thermal degradation of the coated AOS and was the result of the breakage of glucoside bonds and the de-composition of glycosyl units of AOS. The weight loss between 400 °C and 630 °C also indicated that NH_2_ graft modification was successful, and the total weight loss was 61.81%. These results showed that each modification step was successfully carried out, and the MSN-NH_2_-AOS carrier was successfully prepared for Cur encapsulation.

Figure 8b shows the infrared spectra of Cur, MSN-NH_2_, MSN-NH_2_-AOS, and MSN-NH_2_-Cur-AOS nanoparticles obtained using FTIR. MSN characteristic peaks can be observed for MSN-NH_2_, MSN-NH_2_-AOS, and MSN-NH_2_-Cur-AOS, and the absorption peaks at 821, 971, and 1084 cm^−1^ were the three main absorption peaks of MSN. The peaks at 821 and 1084 cm^−1^ were respectively attributed to the symmetric and antisymmetric stretching vibration of the Si-O-Si structure in MSN [55,56]. The bending vibration of Si-OH in the nanoparticles formed an absorption peak at 971 cm^−1^. The absorption peaks at 1405 and 1246 cm^−1^ were caused by ─CH=CH_2_. The peaks at 1632 and 3445 cm^−1^ belong to H-OH or ^−^OH groups on the surface of nanoparticles, indicating that there were numerous hydroxyl groups on the surface of MSN [57]. After combining with AOS, the peaks at the two sites became wider and more obvious, which was caused by the rich -COOH groups in AOS. Due to the existence of different functional groups, the infrared spectrum of Cur showed many characteristic bands. The band at 3445 cm^−1^ corresponds to the ^−^OH, which was related to the tensile vibration of the phenolic hydroxyl group. The bands at 1428, 1281, and 1155 cm^−1^ correspond to the tensile vibrations of some aromatic rings and interfering chains of ketones, consistent with previous reports [58]. The absorption peaks for the characteristic functional groups of Cur were not observed on MSN-NH_2_-Cur-AOS, which also proved that the drug was successfully incorporated into the mesoporous structure of nano-capsules.

XPS can determine the photoelectron binding energy of a sample to infer the composition and structure of the elements on the sample surface [59,60]. We used XPS to analyze MSN, MSN-NH_2_, and MSN-NH_2_-AOS nanoparticles. As shown in Figure 8c, MSN contained Si and O [61]. However, the intensities of Si_2p_ and Si_2s_ peaks were weakened, and the characteristic peaks of C and N elements also appeared, which was mainly related to the polysaccharide coating on the surface. C and N are mainly derived from APTES and AOS, resulting in a decrease in the proportion of the Si element. No N_1s_ peak was observed for MSN in Figure 8d, which shows the N_1s_ spectra of MSN-NH_2_ and MSN-NH_2_-AOS. MSN-NH_2_ shows two peaks for ^−^NH_2_ (401.55 eV) and =N^−^ (400.37 eV), mainly from the grafted amino group, and a distinct peak for ^−^NH_3_^+^ (402.95 eV) appeared for MSN-NH_2_ after AOS packaging, indicating that the modified amino group could absorb the carboxyl group of AOS on the particle surface through hydrogen bonding and electrostatic attraction. In addition, the binding energies of ^−^NH_2_ and =N^−^ peaks were shifted. These results indicate that AOS was successfully coated on the surface of nanoparticles, and MSN-NH_2_-AOS nano-capsule was successfully prepared.

### 3.4. Drug Release and Loading

#### 3.4.1. Drug Release

**Figure 9:**
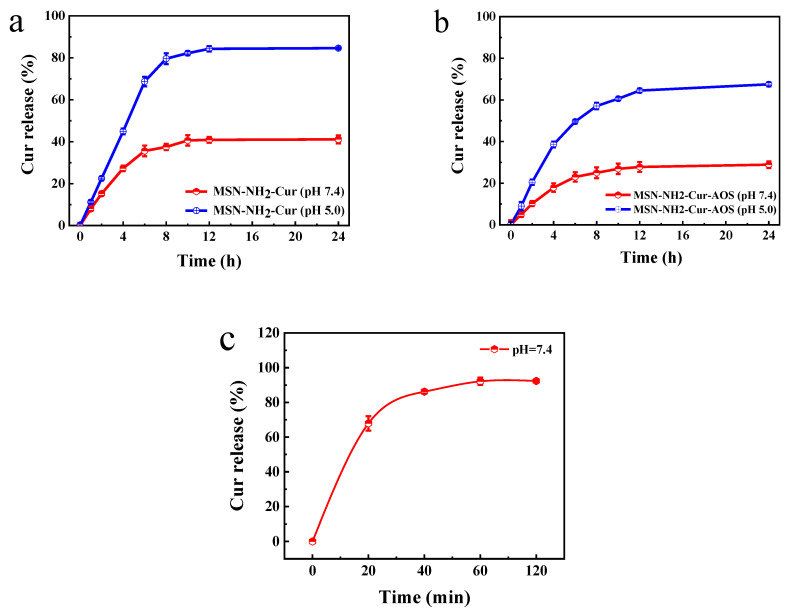
Release rates of Cur from MSN-NH_2_-Cur nanoparticles (**a**) and MSN-NH_2_-Cur-AOS nanoparticles (**b**) under different pH conditions within 24 h. Release rates of curcumin at pH = 7.4 (**c**).

To achieve continuous drug delivery, an appropriate drug delivery system must not only demonstrate appropriate drug delivery efficiency and dosage but also release drugs in a controlled manner [62,63]. The pH in a normal body is 7.4, and the tumor lesions are usually in a slightly acidic state [64]. Therefore, we investigated the release performance of drug-loaded nanoparticles at different pH values. The results showed that the release of nanoparticles was pH-dependent: the release rate is higher at a low pH. As seen from the results depicted in Figure 9a, the cumulative drug release of MSN-NH_2_-Cur in the neutral environment was only about 41.1% and about 84.6% in the slightly acidic environment over a period of 24 h. This may be attributed to the decrease in pH weakening the forces between the drug and the carrier and the effect of the concentration difference between the inside and outside of the nanoparticle. Cur in the MSN-NH_2_ pores diffused outwardly toward the solution and increased its Cur content. Figure 9b shows the curcumin release curve of MSN-NH_2_-Cur-AOS in different pH buffers. In the buffer with pH 7.4, the release rate of Cur slowed down. The cumulative release rate in the first two hours was about 10%, and the total release rate in 24 h was 28.9%, compared with those of the MSN-NH_2_-Cur particles. The release rate in the first two hours was 15.2%, and the total release rate in 24 h was 41.1%. The effect of AOS coating was also studied. When the pH was 5.0, the release rate increased to 67.5% within 24 h. On the one hand, lowering the pH reduced the dissociation of AOS and the electrostatic interaction between molecules. The AOS layer fell off from the surface of the MSN-NH_2_ particle, rendering Cur free to dissociate from the bare internal pores of MSN and diffuse into PBS solution [44]. On the other hand, the difference in drug concentration between the inside and outside of the nanoparticles positively improved the drug release rate. Collectively, these results indicated that the AOS modification could effectively prevent the leakage of Cur from the MSN particle pores and protect the transport of particles in the human body in neutral environments, while the inhibitory effect of the oligosaccharides on Cur was weakened in acidic environments, suggesting that AOS has the effect of a slow release of curcumin. Figure 9c shows that the release rate of Cur can reach 92.3% within 2 h.

#### 3.4.2. Drug Loading

EE and LE are important indexes for assessing the quality of nanomaterials [65,66]. Therefore, we investigated the loading performance of MSN-NH_2_-Cur-AOS and MSN-NH_2_-Cur systems (dosage: 0.25, 0.50, 0.75, and 1.00 mg). The abundant pores of MSN nanoparticles showed superior drug loading efficiency. As seen in Table 4, the LE% of the two groups increased with the increasing Cur doses. With the constant carrier mass, the increase in the Cur dose allowed an easier entry of the drug into the inner cavity due to the concentration gradient between the inside and outside of the carrier. On the other hand, the EE% decreased with the increasing doses of Cur because the amount of Cur exceeded the encapsulation ability of the nanoparticles. The existence of the AOS outer shell was identified as the factor that makes the loading efficiency of the MSN-NH_2_-Cur-AOS particles better than that of the MSN-NH_2_-Cur carrier.

### 3.5. Cytotoxicity

**Figure 10:**
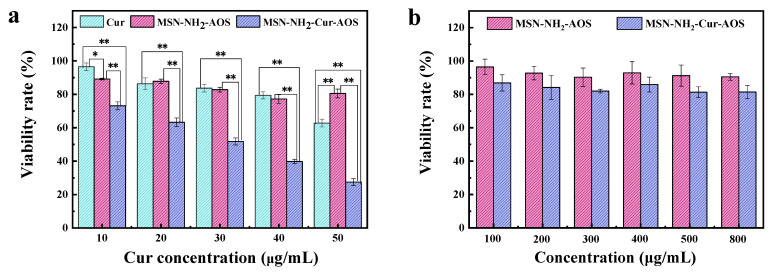
HCT-116 cell viability rates following 24 h treatment with Cur, MSN-NH_2_-AOS (mass of MSN-NH_2_-AOS carrier containing the same amount of Cur), and MSN-NH_2_-Cur-AOS nanoparticles (* *p* < 0.05; ** *p* < 0.01) (**a**). Viability rates of L929 cells treated with different concentrations of MSN-NH_2_-AOS and MSN-NH_2_-Cur-AOS (**b**).

According to literature reports, Cur is safe for normal cells [24,35]. Therefore, we studied the cytotoxicity of the nanoparticles in HCT-116 colon carcinoma cells and the cytotoxicity of MSN-NH_2_-AOS and MSN-NH_2_-Cur-AOS in L929 fibroblast cells using the MTT assay. Figure 10a shows the viability rate of HCT-116 cells after 24 h treatment with Cur, MSN-NH_2_-AOS, and MSN-NH_2_-Cur-AOS nanoparticles. The survival rates of HCT-116 cells treated for 24 h in Cur, MSN-NH_2_-AOS, and MSN-NH_2_-Cur-AOS change in a dose-dependent manner [67,68]. The cell survival rate decreased more slowly compared to the other groups when the cells were treated with free Cur. When the concentration of Cur reached 50.0 µg/mL, more than half of the tumor cells were still viable, with a cell survival rate of 62.76%. This effect was mainly because free Cur does not rely on endocytosis and can only enter the cells by penetrating the cell membrane. When the cells were treated with MSN-NH_2_-AOS at a dose substituting for that of MSN-NH_2_-Cur-AOS carrying 50.0 µg/mL of Cur, the cell survival rate was over 80% and the rate of apoptosis was not significant. The survival rate of cells treated with MSN-NH_2_-AOS was significantly greater than that of those treated with free Cur and MSN-NH_2_-Cur-AOS carrying an equal amount of Cur. At this point, the blank carrier obstructs tumor growth and respiration, which is the major cause of cell apoptosis [69]. Meanwhile, Figure 10b shows that when L929 cells were treated with MSN-NH_2_-AOS at concentrations of 100, 200, 300, 400, 500, and 800 µg/mL, cell viability exceeded 90%. When the L929 cells were treated with the same concentrations as MSN-NH_2_-Cur-AOS, the cell survival rate was greater than 80%. These results imply that the MSN-NH_2_-AOS carrier itself has no cytotoxicity and is relatively safe. Among the experimental groups shown in Figure 10a, the survival rate of the cells treated with MSN-NH_2_-Cur-AOS was significantly lower than that of the other two groups (*p* < 0.01). When the concentration of Cur reached 50.0 µg/mL, the cell survival rate decreased to 27.48%, indicating an increase in cell death in a dose-dependent manner. This shows that the damage to tumor cells mainly resulted from Cur released by MSN-NH_2_-Cur-AOS, and MSN-NH_2_-AOS is a suitable Cur carrier, with no cytotoxicity against tumor cells.

### 3.6. Cellular Uptake

**Figure 11:**
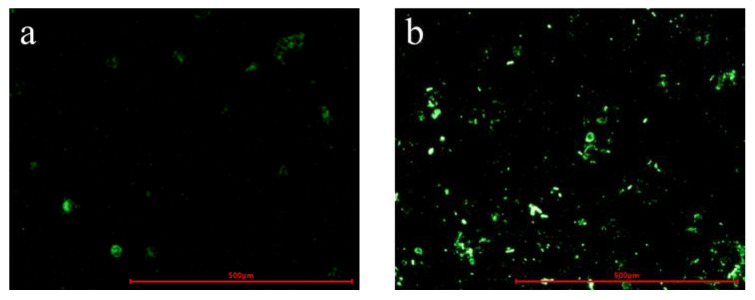
Fluorescence images of cells treated with MSN-NH_2_-Coumarin-6-AOS for 30 min (**a**) and 1 h (**b**).

Cur exerts strong cytotoxicity against a variety of cancer cells. We studied the cellular uptake of MSN-NH_2_-AOS and MSN-NH_2_-Coumarin-6-AOS nanoparticles. Cells treated with MSN-NH_2_-AOS had no fluorescence absorption. As seen in Figure 11a,b, after 30 min of treatment, MSN-NH_2_-Coumarin-6-AOS nanoparticles were partially ingested by tumor cells. After 1 h, the fluorescence intensity was significantly enhanced, and the uptake of drug-loaded nanoparticles by tumor cells increased, indicating that the therapeutic effect was due to the successful absorption of the MSN-NH_2_-Coumarin-6-AOS nanoparticles and release of the drug.

## 4. Conclusions

MSN has the advantages of adjustable pore size and controllable particle size and provides a rich loading space for drugs. Moreover, the presence of large amounts of silicon hydroxyl on the surface provides functionalization sites. In this study, AOS was incorporated into MSN using a silane coupling agent as an intermediate to render the composite nanoparticles pH-responsive, biocompatible and stable. The best AOS:MSN ratio was 1:1, and the average particle size was about 236.8 nm, with a surface potential of −33.1 mV. Below 800 °C, the loss of water adsorbed on the surface of the nanoparticles was only 1.5%, which enables a wide range of applications for drug storage and transportation. The total drug release rate of the MSN-NH_2_-Cur-AOS nanoparticles increased to 67.5% in a slightly acidic environment, and the AOS layer could partially inhibit the release of Cur under neutral conditions (release rate: 28.9%). Our study has shown that the MSN-NH_2_-Cur-AOS nanoparticles loaded with Cur are effective in inducing cellular internalization, and the release of Cur in a controlled manner from MSN-NH_2_-Cur-AOS proposes this carrier as a strong candidate for the tumor-specific delivery of anticancer drugs.

## Figures and Tables

**Table 1:** Particle size, zeta potential, and PDI of MSN-1, MSN-2, and MSN-3.

Sample	Size (nm)	Zeta Potential (mV)	PDI
MSN-1	360.3 ± 3.6	−28.3 ± 0.5	0.356 ± 0.016
MSN-2	390.9 ± 2.6	−29.6 ± 0.3	0.320 ± 0.036
MSN-3	150.8 ± 4.6	−32.2 ± 0.6	0.190 ± 0.039

**Table 2:** PDI of MSN-NH_2_-AOS prepared with different mass ratios of MSN-NH_2_ and AOS.

MSN-NH_2_/AOS (Mass Ratios)	PDI
5:1	0.375 ± 0.01
3:1	0.318 ± 0.04
1:1	0.185 ± 0.035
1:3	0.020 ± 0.025

PDI: Polymer dispersity index.

**Table 3:** The corresponding parameters of N_2_ adsorption and desorption.

Sample	Pore Size (nm)	Pore Volume (cm^3^)	Surface Area (m^2^/g)
MSN	6.165	1.134	783.9
MSN-NH_2_	4.646	1.235	648.1
MSN-NH_2_-Cur-AOS	1.126	0.786	286.3

PDI: Polymer dispersity Index.

**Table 4:** The encapsulation and loading efficiency of MSN-NH_2_-Cur-AOS and MSN-NH_2_-Cur.

Carrier	Dosage (mg)	Curcumin (mg)	EE%	LE%
MSN-NH_2_-Cur-AOS	10	0.25	99.12 ± 0.68	5.01 ± 0.02
	10	0.50	91.24 ± 1.23	9.13± 0.04
	10	0.75	80.74 ± 0.73	12.16 ± 0.10
	10	1.00	74.64 ± 0.49	14.64 ± 0.07
MSN-NH_2_-Cur	10	0.25	88.85 ± 0.65	4.42 ± 0.06
	10	0.50	85.29 ± 0.81	8.52 ± 0.08
	10	0.75	73.29 ± 0.23	11.03 ± 0.03
	10	1.00	70.29 ± 0.23	14.12± 0.09
